# Plant and fungi derived analgesic natural products targeting voltage-gated sodium and calcium channels

**DOI:** 10.1080/19336950.2022.2103234

**Published:** 2022-08-26

**Authors:** Aida Calderon-Rivera, Santiago Loya-Lopez, Kimberly Gomez, Rajesh Khanna

**Affiliations:** a Department of Molecular Pathobiology, College of Dentistry, New York University, New York, NY, USA; b NYU Pain Research Center, New York University, New York, NY, USA

**Keywords:** VGSCs, VGCCs, natural products, plants, fungi, pain

## Abstract

Voltage-gated sodium and calcium channels (VGSCs and VGCCs) play an important role in the modulation of physiologically relevant processes in excitable cells that range from action potential generation to neurotransmission. Once their expression and/or function is altered in disease, specific pharmacological approaches become necessary to mitigate the negative consequences of such dysregulation. Several classes of small molecules have been developed with demonstrated effectiveness on VGSCs and VGCCs; however, off-target effects have also been described, limiting their use and spurring efforts to find more specific and safer molecules to target these channels. There are a great number of plants and herbal preparations that have been empirically used for the treatment of diseases in which VGSCs and VGCCs are involved. Some of these natural products have progressed to clinical trials, while others are under investigation for their action mechanisms on signaling pathways, including channels. In this review, we synthesize information from ~30 compounds derived from natural sources like plants and fungi and delineate their effects on VGSCs and VGCCs in human disease, particularly pain.

**Figure:**
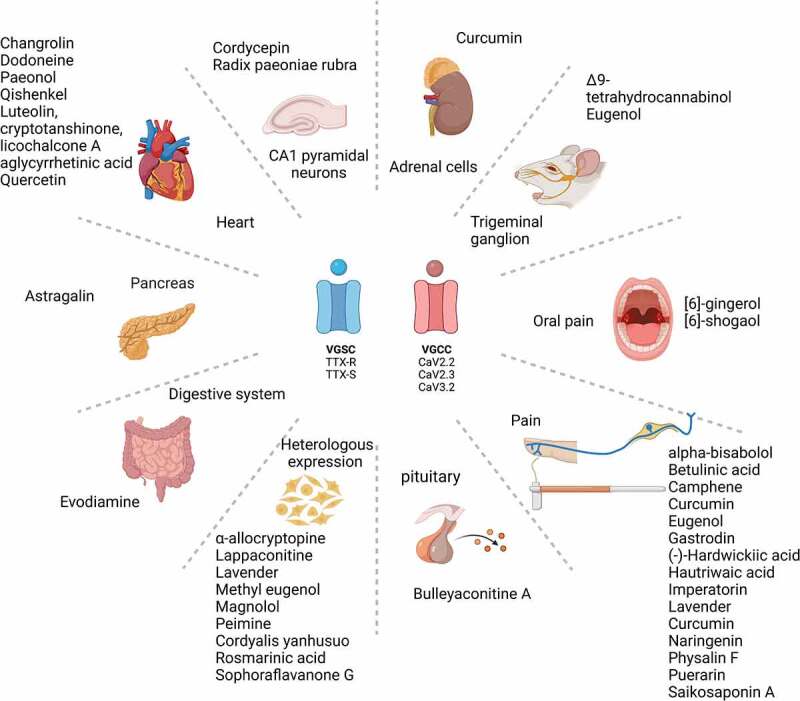

## Introduction

Plants have played an important role in nutrition, recreational usage, and religious practices for as long as we have existed. The first medicinal drugs came from natural sources and existed in the form of herbs, plants, roots, vines, and fungi [1]. Traditional medicine has been used by cultures worldwide, resulting in a generational legacy that helps keep and improve human health [2–7]. Throughout the last two centuries and upon the discovery of the active ingredients morphine (from opium, poppy), salicylic acid (from the bark of the white willow tree), and THC (from cannabis), new chemical analogs have been synthesized to improve their pharmacokinetic properties, avoid toxicity, and decrease side effects [1,8]. The Incas, for example, discovered the effectiveness of local anesthetics (LAs) by using coca-plant leaves to alleviate pain [9]; however, the high frequency of toxicity of cocaine affecting the central nervous and cardiovascular systems prompted the search for more effective and safer LAs. Nowadays, herbal medicines are widely used on up to 80% of the population, consuming them alone or in combination for all kinds of sicknesses [10].

Of the drugs approved by the US Food and Drug Administration (FDA) between 1980 and 2014, 49% were either natural products or derivatives. Natural products represent an important source for the identification of novel drugs [11,12], including those with anticancer and antimicrobial properties, which have been reported to have an action on intracellular and membrane proteins [13–15]. In this review, we present a synthesis of the evidence of action of natural products on two families of transmembrane proteins that play important roles in the electrical signaling of cells – the voltage-gated sodium and calcium channels (VGSCs and VGCCs).

VGSCs and VGCCs are evolutionarily related ion channels that have 25% sequence similarity [16]. Both channel families originated from a common ancestor, the bacterial sodium channel NaChBac [17]. These membrane proteins allow sodium or calcium ions to enter the cell as a result of membrane depolarization [18,19]. They play important roles in action potential generation and propagation, neurotransmitter release, excitation-contraction coupling, and other physiological processes including hormone secretion [18,19]. Although a large number of small molecules have been reported to alter the functional activity of VGSCs [17,20,21] and VGCCs [22,23], the effect(s) and mechanism(s) of action of natural products on these channels are still a matter of investigation. In this review, we begin by presenting a brief overview of the structure and function of VGSCs and VGCCs, and then describe recent contributions (from 2000 until 2021) made in the studies of the effects of plant- and fungi-derived natural products on VGSCs and VGCCs.

## Structure and function of voltage-gated ion channels

### Voltage-gated sodium channels (VGSCs)

The VGSC core is the pore-forming α subunit of 250 kDa, topologically structured in 24 segments arranged in four homologous domains, each one constituted by six transmembrane segments [19]. The symmetric quartet of S1–S4 segments houses the voltage-sensing module. Structurally, this quartet surrounds the pore formed by S5 and S6 [19]. Even though the expression of the α subunit is sufficient for the channel function, one or two β subunits of 30–40 kDa with single membrane-spanning protein topology can join each α subunit and modify their biophysical properties [17,19]. VGSC’s α-subunits are broadly expressed in central and peripheral nervous systems. The family of VGSCs has nine members (NaV1.1–1.9), which can be further divided according to their sensitivity (S) or resistance (R) to tetrodotoxin (TTX). For instance, TTX-S channels, NaV1.1–NaV1.7, are blocked by 500 nM–1 μM of TTX, whereas NaV1.8 and NaV1.9 are resistant to this toxin [19]. TTX-S channels have very fast kinetics, exhibiting fast inactivation that typically occurs within 5–10 ms. NaV1.8 and NaV1.9, however, have slow and ultra-slow kinetics, and produce persistent currents for several hundred milliseconds [19]. In response to membrane depolarizations, activation of VGSC allows current influx through these channels, which contributes to action potential generation and propagation [19].

### Voltage-gated calcium channels (VGCCs)

The central pore-forming α1 of VGCCs subunit holds a transmembrane arrangement like the α subunit of sodium channels [24]. According to their voltage sensitivity, VGCCs are divided into high-voltage-activated (HVA) and low-voltage-activated (LVA) calcium channels [25]. HVA calcium channels (L-, P/Q-, N-, and R-type) are activated at membrane potential more positive than −30 mV. The α1 subunit of HVA channels is associated with up to three distinct classes of auxiliary subunits: the intracellular β subunit; a membrane-associated, disulfide-linked α2δ subunit; and a transmembrane γ subunit [24]. In contrast, the α1 subunit of LVA calcium channels (T-type) does not require ancillary subunits [24]. These are activated by small membrane depolarization that allows calcium entry near the resting membrane potential [26]. VGCCs pharmacological signature profile is unique: dihydropyridines block L-type channels, ω-Conotoxin GVI blocks N-type, ω-Agatoxin blocks P/Q-type, SNX-482 inhibits R-type, while TTA-A2 and Z944 block T-type channels [27,28].

**Table 1.** Composition and source of natural products, their ion channel targets and effects on heterologous cells and neurons, and analgesic actions in vivo.

Natural product	Chemical composition	Source	Target	Model and concentration tested	Ref.
-Not reported-	-	*Allium macrostemon* Bunge Water decoction	↓NaV1.7	HEK cells tested with 50 mg/L. Alleviates pain in Formalin-induced and Acetic-acid-induced pain.	[41]
Alpha-allocryptopine	Alkaloid	*Corydalis decumbens*	↑NaV1.5T353I	HEK cells tested with 30 µM. Rescues NaV1.5 T353I plasma membrane expression.	[44]
Alpha-bisabolol	Terpene	*Cannibus sativa*	↓CaV3.2 ↓CaV3.3 ↓CaV2.1	Inhibited recombinant human CaV3.2 in HEK cells with IC_50_ of 4.5 ± 1.1 μM	[98]
Astragalin	Flavonol glycoside	Roots of *Astragalus membranaceus*	↑L-type current	Rat pancreatic islets tested with 100 µM. Hypoglycemic effect in hyperglycemic rats.	[103]
Betulinic acid	Pentacyclic triterpenoid	*Hyptis emoryi*	↓CaV3.2 ↓CaV3.3 ↓CaV2.2	HEK cells and rat DRGs tested with 20 µM. Alleviates Chemotherapy-induced peripheral neuropathy, HIV-induced sensory neuropathy, and partial sciatic nerve ligation at 2 µg/5 µl.	[104]
Bulleyaconitine A	Diterpenoid alkaloid	*Aconitum bulleyanum*	↓NaV	Pituitary GH3 cells tested with 10 µM. Sensory and motor block of rat sciatic nerve, tested with 0.375 to 0.75 mM.	[46]
Camphene	Monoterpene	*Cannibus sativa*	↓CaV3.2 ↓CaV3.3 ↓CaV2.1	Inhibited recombinant human CaV3.2 in HEK cells with IC_50_ of 7.7 ± 1.8 μM	[98]
Cannabidiol, Δ9-tetrahydrocannabinol	Benzoic acid Diterpenoid	*Cannabis sativa*	↓CaV3.1–3.3	HEK cells tested with 10–30 μM. Mouse trigeminal ganglion neurons tested with 1 µM.	[114]
Changrolin	Phenol	*Dichroa febrifuga* Lour	↓NaV	Rat ventricular myocytes. IC_50_ = 10.19 µM.	[127]
			↓CaV (L-type)	Rat ventricular myocytes. IC_50_ = 74.73 µM.	
Cordycepin	Nucleotide analog	*Cordyceps* spp.(fungus)	↓NaV	Rat hippocampal CA1 pyramidal neurons tested with 80 µM.	[51]
Curcumin	Polyphenol	*Curcuma longa*	↓TTX Nav	Rat DRG neurons tested with 100 mg/kg. Analgesic effect in diabetic neuropathic pain model induced with STZ.	[130]
			↓CaV3.2	Bovine adrenal cells tested with 5 µM.	[129]
Evodiamine	Alkaloid	*Evodia rutaecarpa*	↓L-type CaV	Rat colon smooth muscle cells tested with 10 and 100 µM.	[118]
Eugenol, essential oil	Phenolic acid	Cloves	↓CaV3	HEK cells stably transfected, trigeminal ganglion neurons. IC_50_ = 0.5 mM.	[131]
			↓NaV,	Dental primary afferent neurons. IC_50_ = 0.6 mM Alleviates neuropathic pain in SNL at 50 µg	[29][30]
Dodoneine	Phenolic	*Agelanthus dodoneifolius*	↓L-type CaV	Rat cardiac myocytes. IC_50_ = 10 mM	[115]
Gastrodin	Gastrodia	*Gastrodia*		IC_50_ NaV1.7 = 25.87 µM, HEK cells. IC_50_ NaV1.8 = 67.5 µM, DRGs. Alleviates peripheral neuropathic pain and reverts hyperexcitability.	[56]
[6]-gingerol and [6]-shogaol	Phenolic	Ginger, rhizome	↓NaV1.8 ↓Nav1.1 ↓NaV1.3 ↓NaV1.6 ↓NaV1.7 ↓CaV2.2	IC_50_ NaV1.8 = 45 µM. Analgesic effect in a rat oral ulcerative mucositis model at 300 µM and 150 µM.	[125]
			-	Attenuates pain-associated behavior in rats with SNL at 100–400 mg/kg.	[31]
(-)-hardwickiic acid ((-)-HDA)	Diterpenoid	*Croton californicus* (Euphorbiaceae)	↓NaV TTX-S	Rat DRGs tested with 20 µM.	[57]
			↓NaV1.1, NaV 1.3, NaV1.5	HEK cells tested with 20 µM.	
				Alleviates HIV- and chemotherapy-induced neuropathy at 2 µg/5 µl.	
Hautriwaic acid (HTA)	Diterpenoid	*Eremocarpus setigerus* (Euphorbiaceae)	↓NaV TTX-S	Rat DRGs tested with 20 µM.	[57]
				Alleviates HIV- and Chemotherapy-induced neuropathy at 2 µg/5 µl.	
Imperatorin	Furocoumarin	*Angelica biserrata*	↓NaV1.7	IC_50_ = 28 nM. Analgesic activity in thermal pain and formalin-induced pain in mice.	[32][93]
Lappaconitine	Diterpene alkaloid	*Aconitum sinimontanum* roots	NaV1.5	HEK cells (Acute 30, 60, 100 µM). Irreversible block.	[61]
			↓NaV1.7	HEK cells tested with 30 µM. IC_50_ = 27.67 µM.	[62]
			↓NaV1.3, ↓NaV1.4, ↓NaV1.5, ↓NaV1.8	HEK cells tested with 100 µM.	
Lavender essential oil Silexan, a standardized lavender oil preparation	Monoterpenoid	*Lavender stoechas* and *angustifolia*		Antihyperalgesia in mice with SNI at 100 mg/kg. ↓pERK1/2 and JNK1.	[33]
			↓CaV3.2 ↓CaV2.2 ↓CaV2.1	CHO cells, tested with 1 or 10 µg/ml. Murine synaptosomes and hippocampal neurons tested with 1 µM. Decrease of anxiety-related behavior in humans at 80 mg/d.	[34][35][36]
Linalool essential oil	Monoterpenoid	*Lavender stoechas* and *angustifolia*	↓CaV3.2	HEK cells. IC_50_ = 84 µM.	[138]
			↓NaV	Olfactory receptor cells (ORCs). IC_50_ = 0.56 mM	[133]
Methyl eugenol	Phenylpropene	Asari Radix et Rhizoma	↓NaV1.7	Transfected CHO cells. IC_50_ = 295 μM	[37]
Magnolol	Polyphenolic	Bark of *Magnolia officinalis*	↓NaV	NG108-15 cells. IC_50_ = 15 and 30 µM at −70 and −100 mV, respectively	[38]
			TTX-STTX-R	DRG neurons, TTX-S IC_50_ = 9.4 μM. TTX-R IC_50_ = 7 μM	[78]
Naringenin	Flavonoid	Citrus, tomatoes, and figs	↓NaV1.8 ↓CaV2.2	Rat DRG neurons tested with 100 µM. Reverses mechanical allodynia (pSNL), in males at 30 mg/kg.	[142]
Paeonol	Methoxybenzene	*Paeonia suffruticosa*	↓NaV total	Guinea pig ventricular myocytes. IC_50_ = 17 µM.	[80]
Peimine	Alkaloid	Fritillaria	↓NaV1.7	IC_50_ 47.2 µM	[39]
Physalin F	Steroidal	*Physalis acutifolia*	↓CaV2.2 ↓CaV2.3	Rat DRG neurons tested with 1 µM. Antinociceptive effect on paclitaxel-induced peripheral neuropathy and SNL at 2 µg/5 µl.	[119]
L-Tetrahydropalmatine, protopine, and dehydrocorydaline	Alkaloids	*Cordyalis yanhusuo*	↓NaV1.5 ↓NaV1.7	L-Tetrahydropalmatine, IC_50_ = 7.05 µM. CHO cells stably expressing NaV1.7 and NaV1.5. Analgesic effects in formalin-induced pain model.	[67]
Puerarin	Isoflavonoid glycoside	Kudzu root	↓NaV1.8 through β1 ↓NaV1.7	Rat DRG neurons. IC_50_ = 481.5 µM. Analgesic effect in paclitaxel-induced neuropathic pain at 24 mg/kg.	[85]
Qishenkel Luteolin, cryptotanshinone, licochalcone A, and glycyrrhetinic acid	–	Mixture	↓CaV1.2	Pig cardiac myocytes tested with 0.33 g/kg. Luteolin, cryptotanshinone, licochalcone A, and glycyrrhetinic acid. IC_50_ = 1.5, 5.7, 4.3, and 12.8 µM, respectively.	[40]
Quercetin, Quercitrin, Hyperin, Rutin	Flavonoids	*Acanthopanax senticosus*	↓L-type CaV	Rat ventricular myocytes tested with 283.12 µg/mL.	[121]
Radix paeoniae rubra (paeoniflorin, benzoylpaeniflorin, albiflorin, lactiflorin, oxypaeoniflorin, paeonin, paeoniflorigenone, paenoside, paeonolide, paeonol, galloylpaeoniflorin, gallotannin)	–	Mixture	↓Total NaV	Rat hippocampal CA1 neurons tested with 0.8 mg/ml.	[50]
Rosmarinic acid	Phenolic acids	*Rosmarinic officinalis*	↓CaV3.2	IC_50_ = 53.5 µg/ml.	[138]
Saikosaponins A	Pentacyclic triterpenoid	*Bupleurum Chinese root*	↓NaV1.7	IC_50_ = 28.6 nM. Analgesic activity in thermal pain and formalin-induced pain in mice.	[32][93]
Sophoraflavanone G	Flavonoid	Root of *Sophora flavescens*	↓CaV3.1 ↓CaV3.2	HEK cells, IC_50_ CaV3.1 = 1.4 µM. HEK cells IC_50_ CaV3.2 = 0.75 µM. Alleviates mechanical allodynia.	[43]
Sophoraflavanone G	Flavonoid	Root of *Sophora*	↓HVA	NG108-15 cells IC_50_ HVA = 1.8 µM	[43]
Sophoraflavanone G Vincapusine, Vincarodine, Serpentine	Flavonoid Monoterpene indole alkaloid	Root of *Sophora* Bulbus Fritillaria *Catharanthus roseus*	-	Murine models of inflammatory or neuropathic pain. Inhibits acetic-acid-induced writhing response in the formalin test, paclitaxel-induced neuropathic pain (3 or 1.5 mg/kg p.o.).	[43][124]
			↓CaV3.1	HEK-293 cells transiently transfected. Vicapusine, IC_50_ = 11.83 µM. Vincarodine, IC_50_ = 14.3 µM. Serpentine, IC_50_ = 14.54 µM.	

## Natural compounds with effects on voltage-gated sodium channels (see Table 1)

### Allium macrostemon *Bunge*

The water decoction of the Chinese herb *Allium macrostemon* Bunge is used for the treatment of diarrhea and thoracic pain [41]. Macrostemon crude has been shown to have antinociceptive effects in chemical-induced and heat-induced pain models by decreasing the excitability of dorsal root ganglia (DRG) neurons [41], an important site for pain transmission [42]. This effect can be due in part to the inhibition of NaV1.7 currents [41]. Because the exact components responsible for the analgesic properties of the crude extract are not known, further purification is needed for their identification [41].

### Alpha-allocryptopine

α-Allocryptopine is an alkaloid extracted from *Corydalis decumbens* with anti-arrhythmic properties [43]. In Brugada syndrome (BrS), the loss-of-function mutation T353I in SCN5A, the gene that codes for NaV1.5 channels, causes arrhythmias that lead to sudden cardiac death [44]. α-Allocryptopine has been shown to strongly enhance the peak of the SCN5A-T353I currents and increase the plasma membrane expression of the channels in HEK293 cells. These findings suggest that α-allocryptopine was able to rescue the functional activity of SCN5A-T353I and that the modulatory properties of α-allocryptopine may benefit patients with BrS-associated loss-of-function of NaV1.5 channels [44].

### Bulleyaconitine A

The diterpenoid alkaloid Bulleyaconitine A (BLA) is an active ingredient of *Aconitum bulleyanum* plant that has been approved for the treatment of chronic pain and rheumatoid arthritis in China [45]. The effects of BLA can be explained by its action on neuronal VGSCs. BLA reversibly reduces sodium currents in a use-dependent manner by more than 90% [46]. Interestingly, when injected into the rat's sciatic notch, BLA along with 2% lidocaine or epinephrine to reduce drug absorption by the bloodstream fully blocked the sensory and motor functions of the sciatic nerve [46]. It is noteworthy that this effect lasted for approximately 4 h and returned completely after approximately 7 h with minimal systemic effects [46]. Because BLA strongly reduces sodium currents, it is feasible that various VGSC isoforms in the central and peripheral nervous system are potential targets of BLA [46].

### Cordycepin

Cordycepin is an adenosine and imidazole analog, and the bioactive component of the fungus *Cordyceps* spp. commonly used in traditional Chinese medicine (TCM). Cordycepin has been reported to have a wide range of pharmacological activities, including anti-tumor, anti-bacterial, and anti-inflammatory properties [47–49]. Recently, it was shown that Cordycepin decreases sodium currents in a concentration-dependent manner and causes a 7.4 mV hyperpolarizing shift in the steady-state inactivation of the channels, as well as prolonged recovery time from inactivation. Slower inactivation recovery of VGSCs caused by Cordycepin implies a longer transition of the channels from inactivated to closed states and a lower fraction of the channels available [50]. Inhibition of VGSCs by Cordycepin could, in part, explain the reduced excitability reported in hippocampal CA1 neurons [51], which provides protection against cerebral ischemia and reperfusion injury [52,53]. The mechanism underlying the regulation of VGSCs by Cordycepin is not known; however, Cordycepin can regulate several modulators of VGSCs including ERK [54] and pro-inflammatory cytokines such as TNF-α, IL-1β, IL-6, and IL-8 [49].

### Gastrodin

Gastrodin is the primary bioactive ingredient of Rhizoma Gastrodia, a traditional Chinese medicine used as analgesic in the treatment of chemotherapy-induced neuropathic pain (CINP) [55]. Mechanical and thermal hyperalgesia in rats induced by vincristine were attenuated by intraperitoneal administration of Gastrodin. This compound reversed the hyperexcitability seen in CINP-affected DRG neurons and inhibited the activity of heterologously expressed NaV1.7 channels, as well as NaV1.8 channels in DRG neurons. Gastrodin also reversed CINP-induced overexpression of NaV1.7 and NaV1.8 in DRG neurons. Likewise, Gastrodin decreased the mRNA level of SCN10A, the gene that encodes NaV1.8 channels [56]. These data show that Gastrodin alleviates pain by controlling VGSCs expression and activity.

### (-)-Hardwickiic acid and hautriwaic acid

Native Americans use the fresh leaves of *Croton californicus*, an herb native to the Mojave Desert, to protect against pain [57]. From the screening of a natural products library looking for antinociceptive compounds, (-)-hardwickiic acid ((-)-HDA) from the aerial part of *C. californicus (Euphorbiaceae*) and hautriwaic acid (HTA) from *Eremocarpus setigerus (Euphorbiaceae*) were found to modulate VGSCs in rodent sensory neurons. Using whole-cell patch-clamp recordings, (-)-HDA and HTA inhibited TTX-S sodium currents but not calcium or potassium channels in DRG neurons. (-)-HDA but not HTA blocked NaV1.1, NaV1.3, and NaV1.5 in a heterologous expression system. Neither (-)-HDA nor HTA affected spontaneous excitatory postsynaptic currents (sEPSCs) in substantia gelatinosa neurons of spinal cord slices, indicating that these two natural products preferably target VGSCs over VGCCs. After intrathecal injection of (-)-HDA, this compound attenuated paclitaxel- and HIV-induced sensory neuropathy in rats. Similarly, HTA reversed pain behaviors caused by HIV-sensory neuropathy. The results collectively show the potential for these compounds to inhibit TTX-S VGSCs for pain relief [57].

### Lappaconitine

Lappaconitine is a diterpene alkaloid extracted from *Aconitum sinomontanum* roots. Seventy-six of these species have been commonly used in TCM to treat rheumatoid arthritis, postoperative pain, and cancer pain [58,59]. Intrathecal administration of Lappaconitine into rat spinal cord has shown antinociceptive effects in a model of neuropathic pain caused by chronic constriction injury (CCI) [60]. Its analgesic activity could be explained by the inhibition of NaV1.3, NaV1.4, NaV1.5, NaV1.7, and NaV1.8 VGSCs [61,62]. Lappaconitine has been shown to irreversibly block NaV1.5 channels; however, channels with lysine substitutions within the local anesthetic receptor region at residue F1760 or N1765 are resistant to being blocked by Lappaconitine [61]. These data suggest that by introducing a positive charge within the vicinity of the local anesthetic binding site, disruption of Lappaconitine binding to NaV1.5 channels occurs. It is well established that VGSCs regulate excitability in nociceptive neurons, and they become dysregulated in pain states [63,64]. Therefore, targeting VGSCs with Lappaconitine appears to be a safe strategy for pain relief.

### L-Tetrahydropalmatine, protopine, and dehydrocorydaline

These are active ingredients extracted from the plant *Corydalis yanhusuo* with analgesic, sedative, and anti-arrhythmic effects [65,66]. These derivatives decreased both NaV1.7 and NaV1.5 currents and alleviated formalin-induced inflammatory pain [67]. Among them, Tetrahydropalmatine produced the best analgesic effect [67]. This suggests that VGSCs represent a possible target for these compounds.

### Magnolol

This polyphenolic compound isolated from Houpu, a Chinese herb from the bark *of Magnolia officinalis*, has been reported to have multiple *in vitro* and *in vivo* actions such as anti-inflammatory, anti-oxidative, anti-cancer, vasorelaxant, anxiolytic, antidepressant, anti-nociceptive, and anti-convulsant effects [68–74]. In addition, Magnolol has been reported to exert protective effects against cerebral ischemic injury, memory impairment, and neuronal loss due to aging, hypoxia, and glucose deprivation [75–77]. Magnolol inhibited VGSCs with mild state-dependence in neuronal NG108-15 cells. Moreover, it was recently shown that this compound inhibits TTX-S and TTX-R currents in DRG neurons in a concentration-dependent manner [78]. The blockade of VGSCs by magnolol may explain the reported analgesic effect of this compound in inflammatory pain models in mice [73,74]. Additionally, blockade of VGSCs could be one of the mechanisms leading to the anxiolytic properties of magnolol, given the predominant role of sodium channels in the etiology of anxiety.

### Paeonol

Studies on Paeonol, the main component of the TCM “Mudanpi,” have suggested that this phenolic compound has cardioprotective effects against myocardial ischemia [79]. In order to elucidate its mechanism of action, the effects of paeonol on action potential (AP) characteristics and sodium currents of Guinea-pig cardiac ventricular myocytes were studied. Paenol decreased the AP upstroke phase and shortened the AP duration due to the blockade of VGSCs [80]. These effects were not associated with the blockade of calcium currents nor with the enhancement of potassium currents [80]. These findings suggest that Paeonol, and therefore Mudanpi, may possess antiarrhythmic activity, and can explain in part its cardioprotective effects by selectively targeting VGSCs [80].

### Peimine

Peimine, the main ingredient of Fritillaria – a spring flowering herbaceous bulbous perennial plant in the lily family (Liliaceae), is a pain reliever. In TCM, Peimine is used as a cough remedy that promotes lung dispersing function, is expectorant, and dissolves lumps and masses [81]. In 2016, Jianwei and colleagues reported that Peimine inhibits NaV1.7 heterologously expressed in HEK cells [82]. Some findings suggest that Peimine has a similar blockade mechanism to lidocaine, but the site of interaction remains unknown. It is well known that NaV1.7 activity is upregulated in nociceptive neurons after pain induction, therefore its inhibition underlies analgesia.

### Puerarin

Puerarin is a major isoflavonoid extracted from the Chinese medical herb kudzu root, which has been traditionally used for the treatment of endometriosis, cancer, cardiovascular disorders, and brain injury [83,84]. Puerarin was reported to modulate VGSCs by accelerating their inactivated state, delaying their recovery from the inactivation, and hyperpolarizing their inactivation [85]. Puerarin attenuated mechanical allodynia and thermal hyperalgesia in paclitaxel-induced neuropathic pain by indirectly modulating NaV1.8 channels [85]. Puerarin regulated the functional activity of these channels by targeting their β1 auxiliary subunit known to accelerate the channels’ kinetics [86]. Furthermore, Puerarin also regulates NaV1.7 in a β1-independent manner, by decreasing current influx through these channels [85]. Similarly, Puerarin decreased paclitaxel-induced hyperexcitability in DRG neurons [85]. These findings suggest that the antinociceptive effects of Puerarin could be due to a decrease in VGSC activity that leads to a decrease in neuronal excitability.

### Radix paeoniae rubra *(RPR)*

*Radix paeoniae rubra* (RPR) whose active compounds include paeoniflorin, benzoylpaeoniflorin, albiflorin, lactiflorin, oxypaeoniflorin, paeonin, paeoniflorigenone, paenoside, paeonolide, paeonol, galloylpaeoniflorin, and gallotannin is a natural product mix used in TCM that has been used for treatment of neurological insult [87]. Several studies have suggested that RPR has neuroprotective effects on neuronal damage based on the findings that it significantly suppresses the amplitude of sodium currents in rat hippocampal CA1 neurons, without affecting their current activation, inactivation, or deactivation properties [50]. This effect might predict the protective effect of RPR during brain ischemia seen in clinical trials [88]. The mechanism of action of RPR is poorly understood; however, some studies have pointed out that RPR might activate adenosine A1 receptors [89], leading to the phosphorylation of VGSCs by protein kinase A (PKC) and/or protein kinase A (PKA) [90,91], resulting in an inhibitory action on VGSCs.

### Saikosaponin A and imperatorin

Saikosaponins (pentacyclic triterpenoids), found in *Bupleurum chinense*, and Imperatorin, a constituent of *Angelica biserrate*, have been shown to attenuate neuropathic [92] and inflammatory pain [93]. Saikosaponin A and Imperatorin showed a strong concentration-dependent NaV1.7 current inhibition in CHO cells stably expressing these channels [93]. This indicates that NaV1.7 might be involved in the analgesic mechanism of Saikosaponin A and Imperatorin [93]. Saikosaponins have also been shown to protect against traumatic brain injury and to attenuate neuropathic pain through the inhibition of the p38 MAPK signaling pathway [92,94,95]. Because MAPK can modulate NaV1.7 channels during pain [96], it is reasonable to propose that Saikosaponin A and Imperatorin regulate these channels by a MAPK-dependent pathway.

## Natural compounds with effects on voltage-gated calcium channels (see Table 1)

### Alpha-bisabolol

Recognizing that cannabinoids derived from cannabis [97] have analgesic properties, Gadotti et al. screened eight terpenes that are also found in high concentrations in cannabis plants [98]. The cannabis-derived terpene alpha-bisabolol is present in essential oils and bubble gum products from cannabis cultivars and extracts. Alpha-bisabolol inhibited human recombinant CaV3.2 channels with an IC_50_ of 4.5 ± 1.1 μM with maximal inhibition of ~30%; T-type channels have been reported to be important contributors to nociceptive processing [99,100]. Alpha-bisabolol was similarly effective in reducing human recombinant CaV3.1 and CaV3.3 channels as well as inhibiting T-type channels in sensory neurons from rats. While no effect was observed of alpha-bisabolol on activation of CaV3.2 channels, the terpene shifted the steady-state inactivation curve toward more hyperpolarized potentials, contributing to inhibition of channel activity. Intrathecal administration of alpha-bisabolol reversed licking and biting nocifensive responses to formalin, a model of acute inflammatory pain as well as to complete Freund’s adjuvant (CFA) – a model of chronic inflammatory pain; the latter required CaV3.2 channels as alpha-bisabolol was ineffective in CaV3.2 null mice [98]. Alpha-bisabolol also partially reversed mechanical allodynia in a mouse model of partial sciatic nerve injury [98]. Orally administered alpha-bisabolol was also reported to be effective against visceral pain and in a model of carrageenan-induced inflammation [99]. Thus, the broad analgesic properties of alpha-bisabolol can be attributed to its actions on CaV3.2 T-type calcium channels.

### Astragalin

Astragalin is a flavonoid that has anti-bacterial, anti-fungal, anti-inflammatory, anti-oxidant, anti-ulcer, anti-tumor, and anti-diabetic properties [101]. Pancreatic β-cells act as glucose sensors that detect the amount of glucose in the blood and induce changes in the electrical activity of the cells. This results in intracellular calcium signals that regulate insulin secretion [102]. Astragalin has been shown to increase L-type calcium influx via an ATP-dependent potassium channel mechanism. Intracellular calcium then activates PKC and PKA kinases, leading to insulin secretion in pancreatic islets resulting in hypoglycemia. These findings suggest that by regulating L-type calcium channels, astragalin regulates glucose homeostasis [103].

### Betulinic acid

Betulinic acid (BA) is extracted from the desert lavender plant (*H. emory*) and inhibits KCl-evoked calcium influx in DRG neurons. BA preferentially inhibits transiently expressed CaV3.2 and CaV2.2 calcium channels. BA inhibition of VGCCs leads to a reduction in the frequency of spontaneous excitatory postsynaptic currents (sEPSC) recorded in the substantia gelatinosa, suggesting a presynaptic action of BA. Importantly, BA demonstrated an antinociceptive effect in paclitaxel-, HIV- and nerve-injury-associated peripheral neuropathy. It is well understood that CaV2.2 and CaV3.2 channels play important roles in nociceptive signaling [104]; therefore, pain alleviation by BA could be caused by its effect on VGCCs.

### Camphene

Monoterpene camphene is a pungent-smelling, cannabis-derived terpene present in essential oils. Alpha-bisabolol inhibited human recombinant CaV3.2 channels with an IC_50_ of 7.7 ± 1.8 μM with maximal inhibition of ~25%. Camphene also reduced recombinant CaV3.1 and CaV3.3 currents by ~20% and 10%, respectively, as well as reduced sensory neuron T-type currents by ~20%. Similar to alpha-bisabolol, camphene did not affect activation of CaV3.2 channels but shifted the steady-state inactivation curve by ~4.5 mV toward more hyperpolarized potentials, contributing to the inhibition of channel activity. Intrathecal administration of camphene reversed licking and biting nocifensive responses to both phases of the formalin test. Camphene caused a partial and transient reversal of CFA-induced hypernociception in both male and female mice [98] and also partially reversed mechanical allodynia in a mouse model of partial sciatic nerve injury [98].

### Delta 9-Tetrahydrocannabinol (THC) and cannabidiol (CBD)

Δ9-Tetrahydrocannabinol (THC) and cannabidiol (CBD) are the most prevalent biologically active constituents of *Cannabis sativa*. THC is the prototypic cannabinoid CB1 and CB2 receptor agonist and is psychoactive and analgesic [105,106]. CBD is also analgesic, but it is a CB2 inverse agonist and a weak CB1 receptor antagonist [107]. Interestingly, THC and CBD have been shown to have non-CB receptor-mediated effects in animals including antinociceptive effects in the tail-flick assay of thermal nociception in CB1 receptor knockout mice [108], suggesting some role of other potential targets for their antinociceptive effects. Because T-type calcium channels play an important role in the regulation of nociception, epilepsy and sleep have been reported [109–113], the effects of THC and CBD on the recombinant human CaV3 channels were tested [114]. THC and CBD inhibited all three subtypes of CaV3 channels [114], suggesting that part of the antinociceptive effects mediated by THC and CBD could be due to an effect on T-type calcium channels.

### Dodoneine

*Agelanthus dodoneifoliusis* is one of the medicinal plants used in African pharmacopoeia and traditional medicine for the treatment of cardiovascular diseases. One of the active components, dodoneine (Ddn), is a new dihydropyranone that exerts hypotensive and vasorelaxant effects on rats. Ddn effects have been evaluated in isolated rat heart preparations using Langendorff retrograde perfusion and in freshly dissociated cardiac ventricular myocytes using the whole-cell patch-clamp recording. Ex-vivo, Ddn produces a dose-dependent negative inotropic effect without changing the heart rate. In isolated cardiac myocytes, Ddn reduces L-type calcium current density by ~30%, shifting the inactivation curve toward negative potentials and modifying the half inactivation potentials. Furthermore, Ddn induces a phasic-dependent blocking on L-type calcium currents. These findings suggest that the hypotensive property of Ddn is likely associated with a negative inotropic effect and the blockade of the L-type calcium channels [115].

### Evodiamine

The indole alkaloid evodiamine (Evo) is one of the main bioactive components of *Evodia rutaecarpa*. It has been used for several years in TCM for treating cancer, obesity, inflammation, cardiovascular, and Alzheimer’s diseases [116,117]. The main symptoms of functional gastrointestinal illnesses are motility dysfunction and abdominal pain. The mechanism is related to abnormal changes in smooth muscle and the sensory system, which govern normal gastrointestinal motility. Evodiamine inhibited L-type calcium currents in rat colonic smooth muscle cells (SMCs) as well as spontaneous contractions of the colonic longitudinal muscle strips. Evodiamine’s relaxant effect on colonic motility is caused in part by direct or indirect inhibition of L-type calcium channels [118]. These data show that by acting on L-type calcium channels, evodiamine is useful for the treatment of gastrointestinal motility diseases.

### Physalin F

Physalin F is a steroidal derivative isolated from the herb *Physalis acutifolia* (family: Solanaceae). In DRG neurons, Physalin F inhibits N-type (CaV2.2) and R-type (CaV2.3) calcium channels, without affecting T-, L-, and P/Q type Ca^2+^ currents nor VGSC or potassium currents. Physalin F lowers the frequency, but not the amplitude, of spontaneous excitatory postsynaptic currents (sEPSCs) recorded from laminae I–II neurons in the substantia gelatinosa of the dorsal horn of the spinal cord [119]. The decrease in sEPSC frequency indicates that physalin F inhibits glutamatergic excitatory inputs via a presynaptic mechanism. In complete Freund’s adjuvant (CFA)-inflammatory pain, paclitaxel-, and L5/L6 spinal nerve ligation (SNL)-induced pain models in rats, Physalin F demonstrated antinociceptive effects by targeting VGCCs [119,120].

### Sophoraflavanone G

Sophoraflavanone G is a flavonoid derived from the root of *Sophora flavescens*, which modulates VGCCs. Sophoraflavanone G blocks CaV3.1 and CaV3.2 channels expressed in HEK cells, as well as HVA currents recorded from differentiated NG108-15 cells. Intraplantar administration of Sophoraflavanone G alleviated neuropathic and visceral pain [43], suggesting that Sophoraflavanone G potentially alleviates pain by blocking VGCCs.

### *Total flavones from* Acanthopanax senticosus

*Acanthopanax senticosus* (AS) is a traditional herbal medicine that has been widely used to treat ischemic heart disease. Since VGCCs play an important role in the regulation of cardiac function, the effects of total flavones from AS (TFAS) were tested on L-type calcium currents. Exposure of rat ventricular myocytes to TFAS resulted in a concentration- and voltage-dependent blockade of L-type currents. Moreover, TFAS shifted the activation and inactivation curves of L-type calcium channels toward the hyperpolarizing direction. TFAS significantly reduced amplitudes of myocyte shortening and [Ca^2+^]_i_ with an increase in the time to 10% of the peak and a decrease in the time to 10% of the baseline. Thus, the cardioprotective effects of TFAS may be attributed to the attenuation of [Ca^2+^]_i_ through the direct inhibition of L-type calcium channels in rat ventricular myocytes and consequent negative effect on myocardial contractility [121].

### Vincapusine, vincarodine, and serpentine

Vincapusine, Vincarodine, and Serpentine are monoterpene indole alkaloids extracted from *Catharanthus roseus*. This plant is used in TCM to treat cancer, hypertension, and used as a cardiovascular regulator. It has been reported that CaV3.1 calcium channels play important roles in the regulation of heart’s spontaneous activity of pacemaker cells that generate the cardiac impulse in the sinus node [122,123]. Therefore, these three compounds were tested in HEK cells transiently transfected with CaV3.1 channels. Data showed that Vincapusine, Vincarodine, and Serpentine demonstrated significant inhibitory activity against these channels [124], indicating that the antihypertensive activity of these compounds could be due to inhibition of CaV3.1 calcium channels.

## Natural compounds with effects on both voltage-gated sodium and calcium channels (see Table 1)

### [6]-gingerol and [6]-shogaol

One of the most serious side effects of chemoradiotherapy for head and neck cancer patients is oral ulcerative mucositis (OUM) [125]. [6]-gingerol and [6]-shogaol are two ingredients of Hangeshashinto, a Japanese pharmaceutical-grade drug composed of seven herbal extracts. In an OUM-induced pain model, [6]-gingerol and [6]-shogaol produced lower NaV1.8 currents on cultured rat sensory neurons and in oral mucosal sensation. Preincubation of sensory neurons with [6]-gingerol and [6]-shogaol inhibited veratridine-induced substance P release and action potential generation in a dose-dependent manner. Moreover, [6]-shogaol inhibited currents via NaV1.1, NaV1.3, NaV1.6, NaV1.7, and CaV2.2 [125]. In an oral ulcerative mucositis model [126], [6]-gingerol plus [6]-shogaol in combination with Ginseng extract increased mechanical threshold and alleviated spontaneous pain. These findings suggest that targeting both VGSCs and VGCCs by [6]-gingerol and [6]-shogaol is effective for the treatment of OUM-induced pain.

### Changrolin

Changrolin (2,6-bis[pyrrolidin-1-ylmethyl]-4-[quinazolin-4-ylamino] phenol) is an anti-arrhythmic drug derived from β-dichroine, an active component of the Chinese medicinal herb, Dichroa febrifuga Lour. The molecular basis for its antiarrhythmic effects has been shown to be throughout the inhibition of delayed rectified potassium channels, transient outward potassium channels, VGSC, and VGCC. This multi-current blocking profile observed in rat ventricular preparations seems to lead to a modification of electromechanical function of cardiac myocytes and likely contribute to the termination of arrhythmia [127].

### Curcumin

Curcumin is a polyphenolic compound found in the curry spice turmeric, obtained from the roots of the *Curcuma longa* plant. This phytochemical has been used for centuries in traditional Indian medicine to treat a variety of diseases and conditions ranging from allergies and arthritis to Alzheimer’s disease, diabetes, congestive heart failure, and various malignancies. Studies have demonstrated that curcumin exhibits antioxidant, antifungal, and antitumor activities [128]. Curcumin has been shown to directly modulate the activity of several types of ion channels, such as the cystic fibrosis transmembrane conductance (CFTR) Cl^−^ channel, the inositol 1,4,5-triphosphate receptor calcium channel, and the voltage-gated Kv1.4 potassium channel. It has also been shown that curcumin can inhibit the Adrenocorticotropic-hormone- and Angiotensin-II-stimulated cortisol secretion in adrenal zona fasciculata (AZF) cells potentially via the inhibition of calcium entry through CaV3.2 channels [129]. Additionally, curcumin was demonstrated to effectively prevent and/or ameliorate diabetic neuropathic pain (DNP). This compound decreases the pain threshold (mechanical and thermal) of DNP rats. It was also reported that the increase in TTX-R sodium currents caused by DNP is ameliorated by curcumin [130]. These findings suggest that by acting on different targets, curcumin can relieve pain.

### Eugenol and methyl eugenol

Eugenol, the main phenolic component in the essential oils extracted from cloves, has been extensively used in dental clinics as analgesic. It has been described that eugenol can influence cloned T-type channel isoforms expressed in HEK293 cells. Treatment of trigeminal ganglion (TG) neurons with eugenol inhibited all three isoforms of T-type calcium channels in a concentration-dependent manner. Interestingly, eugenol had little effect on the current kinetics of CaV3.1 and CaV3.2, but did not affect the inactivation kinetics of CaV3.3 channels [131]. Reduction of channel availability enhanced eugenol inhibition sensitivity for CaV3.1 and CaV3.2, but not for CaV3.3. Moreover, eugenol inhibition of T-type channel isoforms was found to be use-dependent. Taken together, these findings suggest that T-type calcium channels are molecular targets for the pain-relieving effects of eugenol [131].

On the other hand, methyl eugenol, a related phenylpropanoid, also used for the treatment of toothache and other types of pain, was shown to preferentially bind to NaV1.7 channels in the inactivated and/or open state [132]. In whole-cell patch-clamp recordings, methyl eugenol tonically inhibited transiently expressed NaV1.7 channels in a concentration- and voltage-dependent manner, suggesting that the antinociceptive and anesthetic effects of this compound could result in part from its inhibitory action on VGSCs.

### Linalool

Linalool is a major component of essential oils such as Jasmine, Rosemary, and Lavender and has been shown to possess various biological effects in the sensory and central nervous systems. Linalool significantly and reversibly suppressed VGSCs in newt olfactory receptor cells (ORCs) as well as membrane voltage-gated currents in newt retinal cells and cerebellar Purkinje cells [133]. The mechanism for this voltage-gated channel block is still unclear, but given that linalool can interfere with the lipids of somatic membranes, this could affect ionic channels directly as some other odorants have been previously reported [134–137].

Additionally, some evidence suggests that linalool also suppresses calcium currents in ORCs, by inhibiting the KCl-induced [Ca^2+^] response of ORCs [133]. Moreover, linalool along with Rosmarinic acid reduces CaV3.2 conductance and shifts its steady-state inactivation when tested on HEK-293 cells. In line with this, no change in the activation properties was observed, indicating that these natural compounds preferentially bind and stabilize CaV3.2 channels in the inactivated state [138]. The inhibition of the T-type Calcium channel CaV3.2 by these natural products may contribute to their neuroprotective and anticonvulsant properties.

### Naringenin

Naringenin is a flavonoid found in citrus fruits, tomatoes, and figs, and has been shown to reduce inflammatory pain in mice by lowering IL-33, TNF-α, IL-1β, and NFκB activation [139,140]. Furthermore, Naringenin inhibits prostate cancer metastasis by decreasing mRNA level expression of SCN9A gene, which encodes NaV1.7 VGSC [141]. Naringenin inhibited depolarization-evoked calcium influx in rat DRG neurons stimulated by acetylcholine, ATP, and capsaicin. This compound was discovered to bind to the VGCC regulator collapsing response mediator protein 2 (CRMP2), in the polar cavity formed by the amino acids P79, Q81, K254, and H198 [142]. In electrophysiology recordings, Naringenin inhibited calcium and NaV1.8 currents in DRG neurons, while also lowered the frequency of sEPSC in substantia gelatinosa neurons. In addition, Naringenin alleviated pain caused by spinal nerve injury (SNI) and reduced postsurgical mechanical allodynia but had no effect on heat-induced nociceptive thresholds. Interestingly, the antinociceptive potential of Naringenin is sex-dependent, as it is only found in males. This data provides evidence that by targeting both VGCCs and VCSCs, Naringenin elicits its analgesic effects.

## Conclusion

Insufficient understanding of the efficacies and adverse reactions of natural products and their molecular mechanisms still represents an important obstacle in modern drug development as well as the clinical translation of naturally derived compounds. Nevertheless, these difficulties can be circumvented by large natural product screening libraries, improved fractionation strategies, and techniques for structural elucidation of active fractions from complex natural product precursors. Together, these approaches afford the accurate exploration of the biological activity of previously inaccessible sources of natural products.

Nature offers a great source of bioactive molecules with activity toward VGSCs and VGCCs. As reviewed in this manuscript, there are many promising drug candidates directed to these channels that have a natural provenance. The present review is intended to show that the chemical diversity of natural products is well suited to provide the core scaffolds for future drugs that may allow the treatment of pathological conditions related to VGSCs and VGCCs malfunction, particularly, but not limited to, chronic pain.

## Data sharing

Data sharing is not applicable to this article as no new data were created or analyzed in this study.
